# Mathematical Model for the Contribution of Individual Organs to Non-Zero Y-Intercepts in Single and Multi-Compartment Linear Models of Whole-Body Energy Expenditure

**DOI:** 10.1371/journal.pone.0103301

**Published:** 2014-07-28

**Authors:** Karl J. Kaiyala

**Affiliations:** University of Washington, School of Dentistry, Department of Oral Health Sciences, Seattle, Washington, United States of America; St. Joseph’s Hospital and Medical Center, United States of America

## Abstract

Mathematical models for the dependence of energy expenditure (EE) on body mass and composition are essential tools in metabolic phenotyping. EE scales over broad ranges of body mass as a non-linear allometric function. When considered within restricted ranges of body mass, however, allometric EE curves exhibit ‘local linearity.’ Indeed, modern EE analysis makes extensive use of linear models. Such models typically involve one or two body mass compartments (e.g., fat free mass and fat mass). Importantly, linear EE models typically involve a non-zero (usually positive) y-intercept term of uncertain origin, a recurring theme in discussions of EE analysis and a source of confounding in traditional ratio-based EE normalization. Emerging linear model approaches quantify whole-body resting EE (REE) in terms of individual organ masses (e.g., liver, kidneys, heart, brain). Proponents of individual organ REE modeling hypothesize that multi-organ linear models may eliminate non-zero y-intercepts. This could have advantages in adjusting REE for body mass and composition. Studies reveal that individual organ REE is an allometric function of total body mass. I exploit first-order Taylor linearization of individual organ REEs to model the manner in which individual organs contribute to whole-body REE and to the non-zero y-intercept in linear REE models. The model predicts that REE analysis at the individual organ-tissue level will not eliminate intercept terms. I demonstrate that the parameters of a linear EE equation can be transformed into the parameters of the underlying ‘latent’ allometric equation. This permits estimates of the allometric scaling of EE in a diverse variety of physiological states that are not represented in the allometric EE literature but are well represented by published linear EE analyses.

## Introduction

Discovering more effective pharmacological and behavioral interventions to counter the burgeoning obesity and diabetes epidemics will require new insights into the biobehavioral regulation of energy balance. Contemporary research in energy homeostasis includes a major focus on the molecular and environmental mechanisms that regulate and modulate energy expenditure (EE) as they pertain to the physiology of energy homeostasis [1], [2], the pathogenesis of common disorders such as obesity and diabetes [3]–[5], and to the identification of new targets for obesity drug development [6]. Progress toward these goals, however, is hindered by problems inherent in how EE is adjusted to account for the differences in body mass and body composition that often confound EE phenotyping [7]–[11].

At present, the standard of practice for adjusting EE for body mass and composition involves linear regression models that adjust EE for one or two body mass compartments, typically total body mass (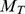), fat free body mass (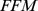) or 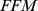 in combination with fat mass (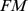) [7], [8], [10]–[12]. Such models typically include non-zero (usually positive) y-intercepts of unknown origin as emphasized in [9]. The non-zero intercept has long been a pervasive topic in discussions of EE analysis, as it both confounds simple ratio-based EE normalization [7], [8], [10], [11], [13]–[18], and stands as an incompletely understood theoretical problem [9]. This problem likely reflects heterogeneity in the mass-specific metabolic rate of individual organs and tissues [9], [19]. Marked heterogeneity in organ-tissue metabolic rates, in turn, has spurred EE analysis into the realm of individual organ-tissue modeling [9], [19]–[21]. This approach again emphasizes linear modeling, and expresses whole body resting EE (REE) in terms of individual organ-tissue masses (e.g., brain, liver, heart, kidneys) and their assumed or estimated metabolic rates per kg of organ mass (mass-specific metabolic rate) [9], [19]–[21]. Multi-organ modeling holds great promise as a tool for addressing a host of problems involving EE regulation and adaptation in health and disease [9]. This approach may also improve model accuracy, and its proponents aver that it might eliminate non-zero y-intercepts [9], [19]–[21].

The ubiquity of linear EE modeling reflects the linearity of EE scaling over modest ranges of body size, a common situation in biomedical research. Over broad ranges of body mass, however, EE scales as a non-linear power function with a zero intercept (allometric scaling) [22]–[26].

Using first-order Taylor series to exploit the ‘local linearity’ of allometric curves, I derive explicit mathematical relationships between the parameters of restricted range linear EE equations and the parameters of the underlying (‘latent’) allometric EE equations. Because allometric scaling also applies at the level of individual organ-tissue REEs [27], [28], I then derive a mathematical model that *1)* reveals how allometric scaling at the level of individual organs and tissues can explain the non-zero y-intercept that typically occurs in linear REE regression equations, and *2)* predicts that linear REE analysis based on individual organs and tissues will not eliminate non-zero y-intercepts. Example instantiations of concepts developed herein involve data that scale EE to a mouse-sized mammal owing to the immense importance of mice in contemporary metabolic phenotyping [11], [29] and my own involvement in this area [7], [8] and http://www.mmpc.org/shared/regression.aspx.

## Materials and Methods

### Single compartment linear EE analysis

Studies designed to identify EE phenotypes typically involve modest within-group ranges of 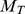 or 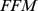. When the range of the body mass covariate is modest, EE typically is well characterized by a linear equation:
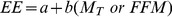
(1)where *b* is the slope parameter (usually positive) and *a* is the y-intercept, which is usually positive in regressions of total or resting EE on 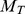 or 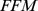.

An important practical aspect of the y-intercept is its confounding effect on the traditional approach to adjusting EE for differences in body size using ratio normalization (dividing EE by 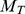 or 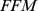) [7], [8], [11], [13]–[18]. The confounding occurs because dividing EE by 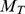 (for example) does not result in a ratio whose expected value is independent of 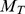 (as so frequently assumed), but rather results in a non-linear 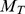-dependent function of the form: 
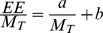
.

Accordingly, whenever the intercept *a* is positive, the “
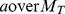
” term decreases in magnitude as 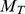 increases such that heavier individuals or groups will appear to be hypometabolic in comparison to lighter ones. This invalidates simple ratio normalization as a method to “remove” the effect of 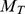 or 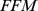 from group comparisons because the ratio tends to be correlated with these mass compartments. This serious problem was first articulated in 1949 by J.M Tanner [13] (also known for developing the Tanner Growth Stage Curves [30]), and has been emphasized in a number of more recent articles [7]–[10], [14], [15].

### Non-linear allometric EE analysis

Contemporary interest in the biological origins of the positive y-intercept [9] in linear EE analysis has evolved in parallel with enduring interest in the biological origins of the value of the scaling exponent *k* in the classical non-linear allometric form of the relationship between EE and 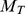 [22]–[28], [31]. Specifically, when 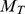 varies over a wide range, EE is well-described by a single power function with a zero intercept:
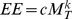
(2)where *k* is a dimensionless scaling exponent that does not depend on the units of EE or 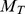, and *c* is a scaling coefficient that does: e.g., if EE is in 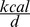 and 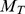 is in kg, then c is in units of 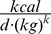.

The scaling coefficient *c* varies widely depending on species, taxa and other factors (e.g., *c* is markedly lower for poikilothermic than for homeothermic animals). The allometric scaling exponent *k* is classically argued to be 0.67 or 0.75 for mammalian “resting”, “basal” or “standard” metabolic rate [22]–[24], [32]–[35], while 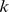 is placed at ∼0.75 for average mammalian field metabolic rate [36]. The theoretical basis of allometric EE scaling remains a topic of enduring interest and controversy [24], [26]–[28], [37]–[42] with explanations for the biological origins of allometric *k* values including those based on the geometric scaling of body heat loss [22], the geometry and physics associated with space-filling fractal circulatory networks [24], and an “allometric cascade” [41], [42]. An alternative mechanistic explanation for 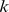 is based on the allometric scaling of individual organ EE with body size [27], [28] (although the explanation for this explanation is up for debate).

Efforts to identify the “correct” exponential 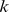 value for basal or REE have been dominated by inter-species analyses encompassing body size over many orders of magnitude [24], [26]. By contrast, little research has been focused on within-species values for 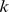. It is known that *k* values for basal or REE vary both among phyla [34], [38] and among mammalian species [43], [44], with values ranging from approximately 0.5 to 0.9 [34], [43]–[45]. Another limitation of this field is that allometric EE analysis has been largely confined to basal or REE, with relatively little attention paid to measures of average 24 h EE [46], [47] or maximal EE [48], despite their indisputable importance to energy homeostasis. Existing research suggests that maximal EE in birds and mammals scales to 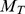 with 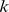 exponents of ∼0.87–0.88 [49], [50]. Finally, with few exceptions [46], [47], [51] allometric scaling has been applied at the level of 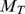 rather than the more metabolically active 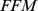 compartment. These considerations highlight the utility of a simple method for estimating allometric scaling parameters from published linear analyses of non-REE outcomes such as 24 h and exercise-related EE, EE during thermoregulatory challenges, and other non-basal states, as well as for EE normalization for differences in body size or composition.

### Derivation of parametric linkages between linear and allometric EE equations

**Figure 1:**
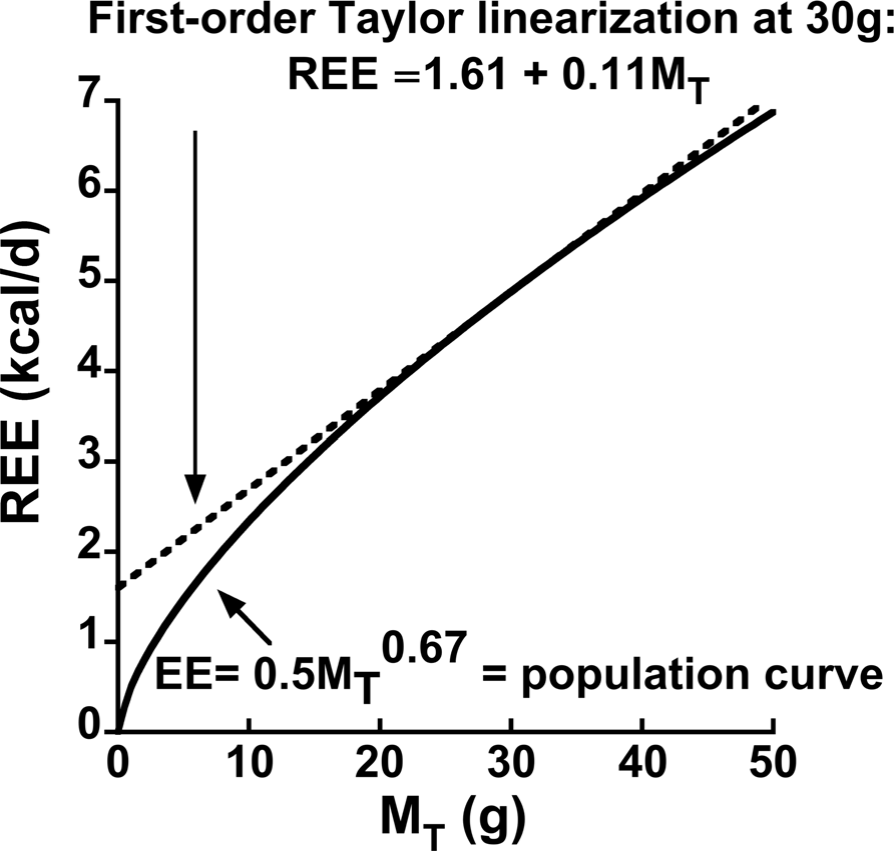
Illustration of a first-order Taylor linearization of a hypothetical population allometric equation for resting energy expenditure (REE): 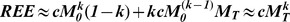 when 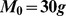, 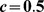 and 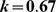. The linearization well approximates expected population REE given total values of body mass 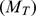 in the vicinity of 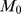. Therefore, given ‘noisy’ sample data with mean 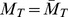, the linear regression model 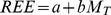 is an estimate of the first-order Taylor series 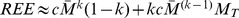 for the true population model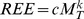.

Because 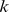 values for basal, resting and average EE typically are less than one, the slope of EE on 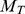 typically decreases with increasing 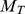 (Figure 1). The evident importance of this fact to positive y-intercepts in linear models of EE has previously been exploited by Wang et al. [52]. These investigators applied a linear regression fit to the errorless allometric curve defined by the Kleiber equation [REE (kcal/d)  =  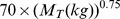] over a restricted range of 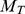 in which the range of 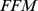 was predicted to be 40 to 80 kg. The analysis revealed that the parameter estimates of the linear regression equation for REE on 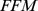 were in good agreement with those of published empirical equations for human subjects [52]. Below I extend this line of thought to an explicit mathematical model that enables one to readily predict the values of the y-intercept and slope parameters of a regression of REE on 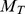 from the scaling coefficient and exponent parameters of the ‘parent’ allometric equation, and vice versa.

Any differentiable non-linear equation exhibits ‘local linearity’ in the vicinity of a particular value of the independent variable. Such equations can be formally linearized in the vicinity of a specific value of an independent variable using a first-order Taylor series [53]: 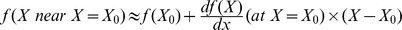, where 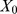 is a specific value about which one chooses to linearize 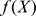. Accordingly, the linearized first-order Taylor series for the allometric REE equation about a particular value of 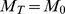 is: 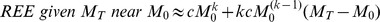. This equation can be rearranged and simplified into the familiar linear equation form having y-intercept and slope parameters as follows:
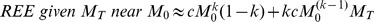
(3)


Taylor linearization is illustrated in Figure 1. The allometric curve shows the trajectory of REE based on Eq. 2 with a scaling exponent of 0.67 [22], [33], and a scaling coefficient of 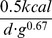, a based on an analysis of normal chow-fed mice [35]. Now imagine that the curve in Figure 1 represents the mean of REE given 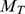 for a population of small mammals.

Note that the straight line for REE based on the first-order Taylor series nearly overlies the population non-linear population allometric curve across a substantial range of 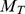 (∼23 to 29 g) such that this curve exhibits a substantial breadth of local linearity. It is apparent, therefore, that a straight line which is tangent to a population allometric curve at a particular value of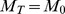 is precisely equivalent to the first-order Taylor formula for that population curve at that 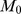. Therefore, a linear regression model that is fit to sample data with mean 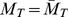 from a population in which the allometric model is true has the following interpretation: the regression model 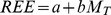 is an estimate of the first-order Taylor series 
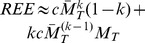
 for the true population model 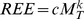. The accuracies of 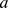 and 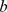 as estimates of 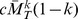 and 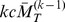, respectively, will depend upon the sample size, the r-square of the linear fit, the range of 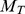 and other variables. Note that I do not claim that these are unbiased estimates in a formal statistical sense, only that they are ‘reasonable’ estimates (for confirmation see Appendix S1).

We can now estimate the allometric scaling exponent 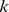 and the scaling coefficient 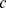 from the 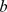 slope and 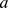 intercept parameters of a simple linear fit to an available but incomplete range of 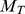.

We form the following system of equations:
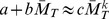
(4)

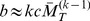
(5)


Dividing Eq. 4 by Eq. 5, rearranging and inverting yields:
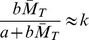
(6)


Solving Eq. 4 for *c* yields:
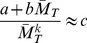
(7)


Appendix S1 presents a proof of concept to the effect that *1)* the restricted range regression model 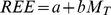 is a ‘reasonable’ estimate of the first-order Taylor series 
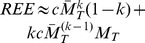
 for the true population model 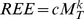 and *2)* that the new method using Eqs. 6 and 7 does provide ‘reasonable’ estimates of 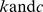.

### Model for the positive y-intercept in linear regressions of EE on body size

A number of energetics researchers have been developing models of whole-body REE based on the concept that total REE equals the sum of the individual tissue-organ REEs [19], [20], [27], [28], [54], [55]. Particularly germane to my paper are the conceptual framework and data analyses by Wang et al. [27], [28]. Their work indicates that allometric scaling pertains at the level of individual organ-tissue REEs, and that allometric scaling at that level can serve as a basis for whole-body allometric REE scaling [27], [28]. Specifically, Wang et al. modeled whole-body REE as the sum of four high mass-specific REE organs (liver, brain, heart and kidney) that collectively account for ∼60% of whole-body REE, and one low mass-specific REE compartment termed ‘remaining tissues’, calculated as 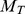 minus the sum of the four high mass-specific REE tissue masses [27], [28].

The basic model put forward by Wang et al. [27], [28] is:
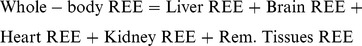
(8)


Wang et al. analyzed a large body of published data to model *1)* the manner in which the mass-specific REEs of individual organ-tissues scale with 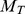; and *2)* the manner in which the individual organ masses scale with 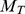. This allowed the REE of each organ-tissue in Eq. 8 to be modeled as the organ’s mass-specific REE × the organ’s mass, where mass-specific REE and organ mass were each based on allometric scaling to total body mass 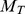 [27], [28]. The mass-specific REE for a particular organ-tissue has the allometric form 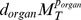, and the organ mass for that particular organ-tissue has the form 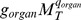. I stress that the 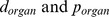 parameters scale the organ’s mass-specific metabolic rate to total body mass, 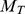, while the 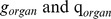 parameters also scale the organ’s mass to 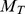._Accordingly, the REE of a particular organ, scaled to 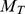, is: 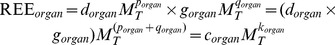.where 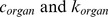 represent the lumped parameters that scale the individual organ’s REE to 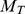.

This construction allows the general form of the equation for REE to be expressed as a sum of power functions each of which is expressed as a function of 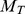:
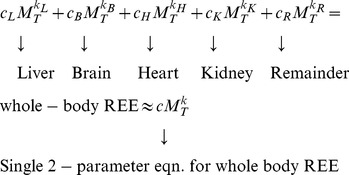
(9)


The “approximately equal to” sign needs explanation. This formality is necessary because a sum of power functions does not, in general, *exactly* equal a single 2-parameter power function across the range of the independent variable (the correspondence can be very close, as discussed below). As such, no straightforward analytic way exists by which the parameters on the right hand side of Eq. 9 can be identified from the parameters on the left hand side. Instead, cumbersome approximation methods have been developed for this class of problems, such as the Prony’s sum of exponentials method [56]. (The present analysis leads, however, to a simple approximation method as discussed below).

Wang and co-workers [27], [28] formulated the concept expressed by Eq. 9 to assess the validity of “Kleiber’s Law” [23], [32], which holds that the mass scaling exponent *k* on the right hand side of Eq. 9 equals 0.75 when the dependence of REE on 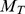 is analyzed over many orders of magnitude (a point that has been debated intensely for more than 70 years). My goal is to document that when linearized using Eq. 3 the model in Eq. 9 leads to an explanation for the source of the positive y-intercept in linear regressions of EE on 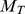 or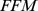.

Specifically, given any value of 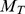 in the vicinity of 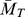, an organ’s REE can be expressed as: 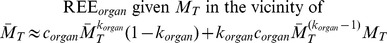.

Summing the individual organ REEs allows us to express whole-body REE given a value of 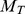 in the vicinity of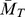 as the sum of the individual organ-tissue Taylor intercept + slope × 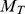 terms as follows:
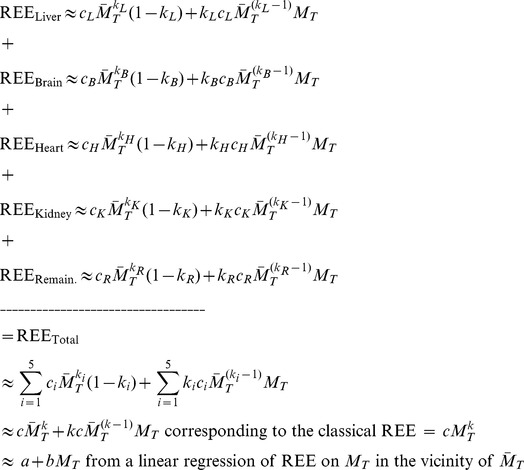
(10)


where the *i* subscript indicates summation over the five organ-tissue compartments.

**Figure 2:**
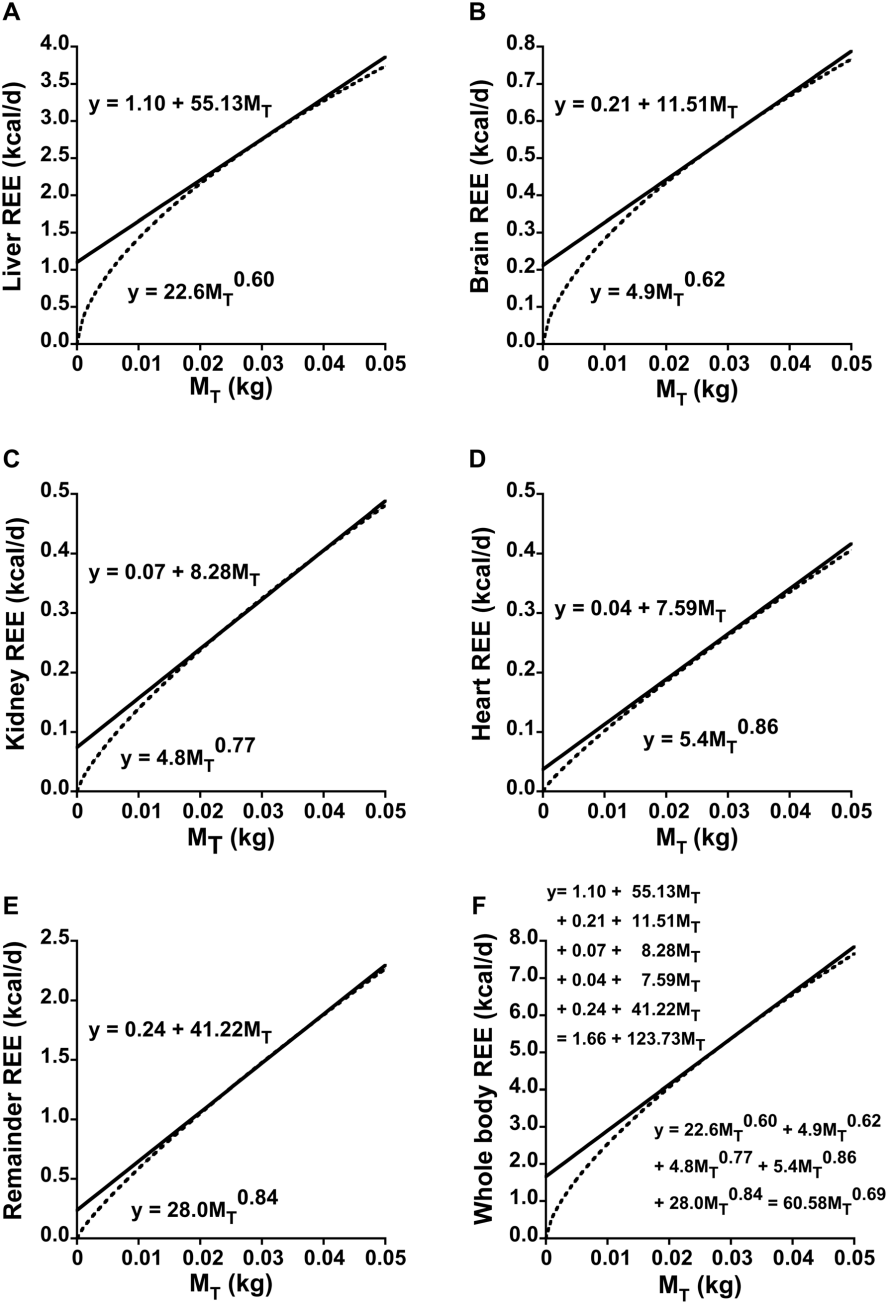
Instantiation of the model for the contribution of individual organs and tissues to the y-intercept in linear regressions of resting energy expenditure (REE) on total body mass (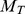). Panels A through E depict individual organ-tissue contributions to REE scaled to 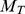 in accordance with the approach and numerical values for allometric scaling coefficients units expressed in 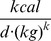 reported in [27]. Note that the y scales differ. The REE for each organ-tissue is expressed as a first-order Taylor linearization at a specific body mass 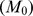 of 0.03 kg (upper equation) of the parent allometric function (lower equation). Panel F reveals that the sum of the linearized equations equals total REE at 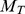 = 0.03 kg and very nearly equals total REE in the range 0.02≤ 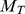 ≤0.04 kg. The aggregate y-intercept (1.66) is the sum of the individual organ-tissue y-intercepts, while the aggregate slope (123.73) is the sum of the individual slopes. Note the particularly large contribution to the y-intercept and to whole-body REE by the liver even though it represents only ∼5% of 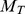. Applying Eqs. 6 and 7 with 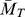 = 0.03 kg in the aggregate linear equation results in the parameters 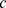  = 60.58 and 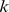  = 0.69 of a single 2-parameter allometric equation for the whole-body EE curve. These parameter values are remarkably similar to those identified by standard log-log analysis or by non-linear regression (see text). To convert the units of a scaling coefficient to 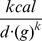, divide by 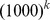. To convert the slope of a Taylor series to units of 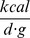, divide by 1000; the intercept remains unchanged.

An instantiation of Eq. set 10 is depicted in Figure 2 based on numerical values for the parameters of the five organ-tissue allometric terms from [27]. Reference [28] gives similar values.

### Implications for EE analysis at the individual organ level

Advances in technology for imaging and quantifying organ mass have made it possible to develop linear models whereby human REE is expressed as mass-specific REE-weighted sums of individual organ masses [9], [19], [20], [55]. Such models may have advantages over current approaches that model REE in terms of one or two-compartment linear functions. In particular, one of the major goals of EE analysis is to more accurately estimate or explain EE than do established modeling approaches that do not account for the heterogeneity of the organ-tissue proportions and mass-specific EEs in the 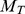 and 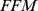 compartments. At present, multi-organ models are limited to analysis of REE in humans, but this approach likely will be extended to animal models and to other measures of EE [9].

Major proponents of organ-tissue level EE modeling have argued that: “Not only might accounting for organ energy expenditure reduce between-subject variability in REE, but it also might allow REE to form a ratio to body composition that is independent of body size [9]”(p.13). Some published multi-organ models for REE in humans do lack significant y-intercepts [9]. My analysis can be extended, however, to suggest that eliminating the y-intercept may not be a reliable consequence of multiple organ-tissue models of REE.

As an example, let 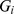 denote the mass of a particular organ-tissue. If 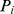 is the proportion of 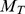 that the organ represents, then at 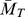, 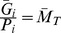. Accordingly, for each organ-tissue in Eq. 10:
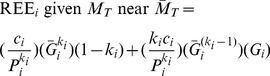
(11)


Eq. 11 predicts the parameters of a multiple linear regression equation for REE based on individual organ-tissue masses. Each beta coefficient (slope parameter) for organ mass is an estimate of 
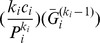
, and the y-intercept is an estimate of the sum of the 
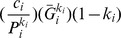
 terms.

### Quantitative analysis of the importance of scaling coefficients and exponents to the y-intercept

Organs with large 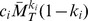 [or, equivalently, 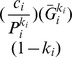] intercept values are predicted to strongly influence the magnitude of the y-intercept. Note that the ‘
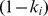
’ factor is critical because if the individual organ-tissue REEs scale with 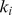 values that are less than unity, then the model predicts that accounting for individual organ masses will not make the aggregate y-intercept term equal zero. Rather, achieving a zero y-intercept would require that the REE of one or more organ-tissue components scales with a 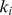 of greater than unity, which seems unlikely based upon interspecies analyses [27], [28]. This prediction is contrary to the sense that organ level REE analysis might result in prediction equations that lack y-intercept terms [9], [57].

It should be noted, however, that the dependence of an intercept term on 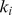 is made more complex because 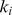 also functions as an exponent. Accordingly, I explored this issue more thoroughly to gain further insight.

**Figure 3:**
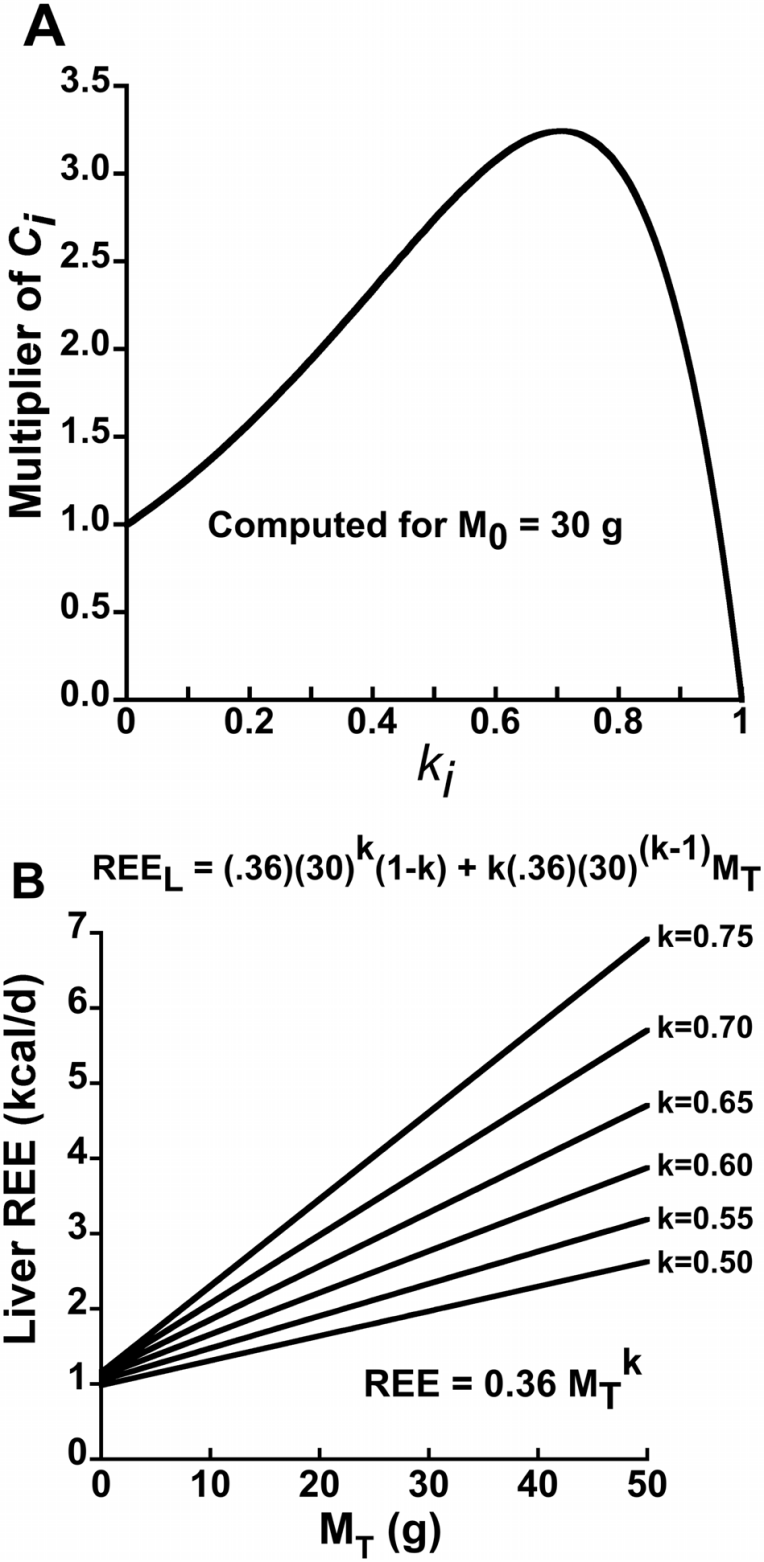
Influence of the organ-tissue scaling exponent 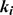 on an organ-tissue’s contribution to the positive y-intercept and to REE in linear models. Panel A depicts the contribution to the y-intercept by the *i-th* individual organ-tissue REE in terms of 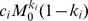, where 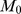 is the value of total body mass about which the Taylor linearization is performed. The contribution to the y-intercept is expressed as a multiplier of 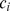 calculated as 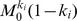. The individual organ-tissue’s contribution to the y-intercept is maximized given a fixed numerical value of 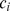 when 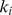 is ∼0.70 for an animal with 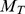  = 30 g, as predicted by Eq. 12. Importantly, there is a substantial range of 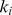 values that more than double 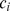. Panel B depicts the hypothetical effect of varying the 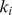 value on both the y-intercept and slope of the hypothetical liver REE – 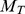 relationship assuming that the allometric scaling coefficient 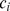 remains fixed at 0.36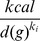 (rescaled from 22.6 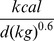, the value reported by [27] and depicted in Figure 2A). Note that the sensitivity of the slope to variation in 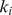 suggests that group differences in the 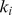 of the liver, a small organ with a big impact on whole-body REE, could contribute to the problem of differing between-group slopes of whole-body REE in phenotyping studies.

Based on elementary calculus, the function 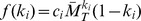 achieves a maxima when 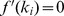. Note that 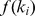 simply equals the allometric scaling coefficient 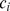 times a multiplier, 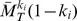. Taking the derivative of 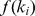, setting it equal to zero and solving for 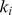 reveals that the maximum value of the multiplier occurs when:
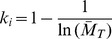
(12)where 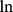 denotes the natural log. If 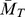  = 30 g, then the multiplier achieves a maxima at 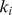  = 0.706, as depicted in Figure 3. Note that the scaling coefficient multiplier more than doubles 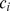 over a fairly broad range of 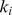. Accordingly, a small organ can have a marked influence on the size of the y-intercept if the lumped allometric equation for that small organ has a big scaling coefficient (which reflects a high mass-specific REE) and a mass scaling exponent 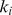 value that is only moderately less than unity as depicted in Figure 3. This analysis has implications for explaining why the slopes of EE on 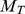 or 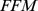 might differ between groups (Figure 3B and discussion).

## Discussion and Conclusions

In a 2012 review [9] Heymsfield and colleagues wrote: *“The distinguishing feature of [linear] statistical REE-body composition models is a non-zero intercept of unknown origin”* (p. 13). The present analysis provides a testable model for the intercept’s origin (Eq. 10 and Figure 2).

This model is premised on the local linearity of the more fundamental non-linear allometric scaling of EE. The Taylor series formalism for local linearity was the key to revealing the mathematical relationships between the parameters of restricted range linear EE equations and parameters of the ‘latent’ allometric EE equations (Eqs. 6 and 7). To my knowledge, these mathematical connections had not been recognized previously despite many decades of interest and analysis devoted to allometric and linear EE models. This shared parameterization and the concept that allometric scaling applies to individual organ-tissue REE then led to Eq. 10.

It should be stressed that the conceptual validity of Eq. 10 is not dependent upon particular published values of the parameters that define the allometric scaling of individual organ-tissue REE with 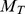. Nor does the model’s validity even require the mass-specific REEs of individual organ-tissues to vary as allometric functions of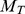 within species, an important point given the need for more research on this topic (some research suggests that within-human mass-specific organ-tissue REE is relatively constant across body size in healthy adults [58]–[61] but is higher in children and lower in the elderly [62], [63]). Indeed, efforts to refine our understanding of the within species scaling of individual organ-tissue REE to 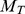 (and to 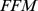) likely will be an important focus of future research [9]. Data more firmly indicate that organ-tissue mass does scale with 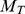 [9], [55], an important consideration for the validity Eq. 10. The numerical scaling values from [27] that are presented in Figure 2 simply permit a concrete example of Eq. 10. Note also that the compartmental model of Wang et al. [27], [28] is but one of many possible models. Indeed, an earlier model encompassing a greater number of individual organ-tissue terms was published by Wang et al. [52]. The model utilized herein was chosen because it parsimoniously captures the major contribution of the high metabolic rate organs to whole-body REE [9], [27], [28].

The preferential focus of EE analysts on REE reflects its enduring status as a metabolic construct because it represents the majority of total 24 h EE (60–75%), is highly correlated with 24 h EE, and yet can be quantified in a relatively short time interval [9]. However, it is important to stress that linear univariate and multiple regression analyses involving average 24 h EE also typically involve non-zero (usually positive) y-intercepts [7], [8], [64]. Hence, the form of the model developed for REE should extend to 24 h EE as well.

It had been hypothesized previously that non-zero intercepts in linear REE models reflect marked differences in the mass-specific metabolic rates of the individual organs and tissues that make up the metabolically active tissues of the body [9], [19], [27], [65], [66]. Taken at face value, this concept implies that accounting for individual organ-tissue metabolic rates in linear EE modeling might eliminate significant intercept terms, and indeed some linear models involving individual organ-tissue REEs do not have significant intercepts [9], [19], [20]. My analysis predicts, however, that zero intercepts will not be upheld as a reliable feature of individual organ-tissue REE linear modeling. This prediction depends, however, on the key assumption that REE at the individual organ-tissue level scales to 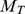 with allometric exponents of less than unity. The extent to which this premise holds in humans is unclear. However, the fact that linear regressions of human REE on 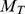 or 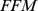 typically involve substantial positive y-intercept values [9], [64] is certainly congruent with the hypothesis that human individual organ-issue REE scales to 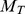
(and to 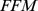) with allometric scaling coefficients that are less than unity.

Multi-organ-tissue EE analysis will be adopted on a more widespread basis in human research, and likely will be developed into a vital tool in basic animal energetics research [9]. This approach has been motivated, in part, by the fact that the sum of brain, kidneys, heart and liver account for the majority of REE despite their small aggregate mass. For example, in humans, ∼60–70% of REE is generated by these four organs even though they make up only ∼6% of 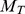 (reviewed in [9]). In small mammals, the proportional contribution may be even larger, and this issue is of considerable importance given the major role of mice in studies on the molecular mechanisms that regulate energy expenditure [8], [11].

### Implications for murine EE phenotyping and for group comparisons when the slopes of EE on body size are not homogeneous

Given that the liver is predicted to contribute ∼51% of total REE and ∼67% of the total y-intercept in a 30 g mouse based on the parameter estimates in [27], an intriguing hypothesis follows: Genetic, pharmacological, nutritional or pathological factors that alter liver metabolism could have marked impacts on whole-body REE and its assessment in murine EE phenotyping. Consistent with this hypothesis, whole-body REE adjusted for lean mass is elevated by ∼5–10% in humans with type 2 diabetes [67]–[72] owing to the increased metabolic cost of elevated hepatic glucose production (HGO) [68], [71], [72]. Considering that liver REE represents “only” ∼17% of whole-body REE in a 70 kg human (based on the allometric equations depicted in Figure 2), it would seem that the impact of elevated HGO on whole-body REE in mice with experimental or acquired diabetes could be substantial indeed. Similarly, naturalistic homeostatic challenges that alter whole-body metabolic rate in mice (e.g., thermal stress [1] or food intake) might do so, in part, via effects on liver EE. A supportive finding is that mice selected for high food intake exhibited significantly higher REE compared to mice selected for low food intake, and greater liver mass was the dominant morphological trait associated with the elevation in REE [73].

A key assumption of ANCOVA, the standard of practice for adjusting EE for a body size covariate [7], [8], [10]–[12], is that the slope of EE on the covariate is the same for each group being compared. Accordingly, ANCOVA fits a single (“pooled”) slope estimate to the data. In some instances, however, a statistical test of the equal slopes assumption leads one to reject it, in which case separate slopes can be fit to the groups using an extension of ANCOVA [74] (for examples and more information see http://www.mmpc.org/shared/regression.aspx). This complicates both the analysis and its interpretation, but perhaps more importantly raises a fundamental and poorly understood question: why does the EE vs. body size relationship differ between groups? While it is easy to speculate regarding a mechanism (e.g., in the leaner of two groups being compared each unit increase of 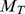 might be expected to contribute a relatively greater increase of EE), this issue likely represents a complex problem owing, for example, to potential EE regulatory effects via adiposity-related negative feedback signaling [7]. My analysis suggests a novel potential mechanism for unequal slopes, and again it involves the liver. Specifically, group differences in the allometric scaling of the liver can, hypothetically, promote group differences in the slope of the whole body REE - body size relationship (illustrated in Figure 3B). At 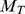  = 30 g, a 0.05 unit change in 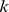 is predicted to change liver REE by more than 15%, which translates to a ∼9.5% increase in whole-body REE if 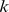 is simply changed from 0.6 to 0.65, yet the y-intercept remains almost unchanged in accordance with Figure 3A (calculations assume that the numerical value of 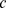 is held constant at 0.36, see Figure 3B). It should be noted that the ‘remainder’ compartment is also positioned to affect the slope of EE on 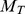 because it contains skeletal muscle and storage fat, and because this compartment accounts for a substantial proportion of whole-body REE (∼27% in mice based on the parameter estimates depicted in Figure 2E). Thus, one might expect that the allometric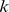 exponent for the remainder compartment would be lower in obese compared to lean individuals, but this prediction assumes that metabolic signals that are secreted in proportion to fat mass (e.g., leptin) do not influence the intrinsic metabolic rate of skeletal muscle or other constituents of the remainder (or other) compartment(s) (a dubious assumption [75]). Note that while the estimates presented herein regarding the importance of the liver to whole-body REE rely on the individual organ-tissue REE parameter estimates presented by Wang et al. in [27], similar results are obtained when one uses the parameter estimates in related work by Wang et al. [28]. In particular, the latter analysis predicts that the liver and remainder compartments account for ∼47% and 30%, respectively, of whole-body REE in mice.

### EE normalization to metabolic body size

How best to adjust EE for body mass and composition in basic animal research has been a topic of sharply renewed focus in recent years [7], [8], [10], [11], [76]. The ability to easily estimate 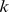 using Eq. 6 has a practical application for adjusting EE to control for differences in body size. A classical method for EE normalization is to divide EE by body mass raised to the appropriate allometric scaling exponent [23], [32], [37], [45], [77] (inspection of Eq. 2 shows that 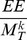 equals a constant). Indeed, some sources term the exponent a “normalization constant” [78]. The quantity 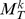 is a measure of ‘metabolic body size’ [23], [32], where this construct is defined as the *“…body size which is chosen so that the metabolic rate per unit of this body size is the same for large and small animals*” [32] (p. 512). In the mid-1950s, Dobeln argued that a better measure of metabolic body size is the adipose tissue free component of body mass (similar to FFM) raised to an exponent [51], [79], but the concept of normalizing EE to 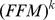 has been translated into practice in just a few studies [46], [47].

Importantly, in experimental work employing the metabolic body size approach for EE normalization, the choice of the scaling exponent was based on an assumed value [77], [79], [80], yet clearly the exponent is not an immutable constant [26], [38], [81]. Therefore, obtaining an empirical estimate of 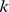 for any given data set would permit the formulation of more valid ratios for group comparisons. Specifically, one could divide each animal’s EE by its M or FFM raised to the 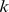 value computed in accordance with Eq. 6. This method (or the classical method based on the regression of log (EE) on log (M) [81]) will result in a normalized EE construct that is largely or completely uncorrelated with body size. Nonetheless, regression-based methods such as analysis of covariance will remain the preferred option in analyses designed to infer EE differences between groups [7], [8], [10]–[12], [15], [81].

### Estimating allometric parameters from published linear equations

Eqs. 6 and 7 permit one to readily estimate allometric equation parameters from any of a vast number of published human and animal studies that provide only linear fits of EE on 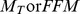, and which involve a diverse variety of metabolic states that are poorly represented or completely absent within the existing allometric literature (e.g., 24 h EE; exercise; diabetes; overfeeding; underfeeding; genetically altered mouse models). For example, a classic paper by Ravussin et al. [64] involving particularly rigorous measurements of EE in n = 177 humans of widely varying adiposity (% body fat ranged from 3 to 50%) presents the following linear equation: 24 h EE in 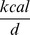  = 597+26.5 FFM, r^2^ = 0.81. Using Eqs. 6 and 7 this translates to: 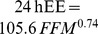. Accordingly, one obtains an indication that adult human 24 h EE scales to FFM with an exponent that is similar to the classic Kleiber value of 0.75 [23].

### Estimating the parameters of a classical 2-parameter allometric equation from a sum of allometric equations

In Eq. 9, existing methods for linking the 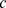 and 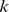 scaling parameters of the right hand classical allometric equation to the parameters of the five individual allometric functions on the left hand side rely on complex estimation procedures such as Prony’s sum of exponentials method [56]. The simplest practical approach (and used by Wang and co-workers [27], [28]) is to computationally sum the allometric functions for REE on the left hand side over the desired range of 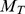, and then fit a 2-parameter allometric equation to the resulting REE sum. The present work reveals another option: simply linearize each REE term, sum the y-intercepts and slopes to obtain the aggregate linear equation (Eq. 10, Figure 2), and then use Eqs. 6 and 7 to identify the scaling coefficient and scaling exponent of the 2-parameter allometric equation. In the example depicted in Figure 2, this method yields the allometric equation of 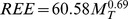 with a residual sum of squares of 0.0106. For comparison, the equation based on a classical regression of natural log (REE sum) on natural log (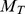) is 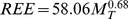 and has a 2.4-fold larger residual sum of squares of (0.0259), while the equation based on nonlinear regression of the sum on 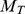 gives 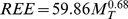 with a 47% smaller residual sum of squares (0.0056) than the new method. Although each of the three methods provides an excellent fit to the curve, linearizing each term of a sum of power functions provides a particularly simple solution to the problem of translating the parameters of individual power functions to the parameters of a single 2-parameter power function for Y in a region of X that is of particular interest. It should be stressed that the value of the allometric scaling exponent does depend on the range of 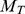 over which the analysis is undertaken, a point with a provenance in a 1982 analysis by Heusner [35]. Indeed, when analyzed over a range of 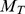 that spans many orders of magnitude, the individual organ-tissue allometric parameters [27] that were used above to arrive at allometric scaling exponents of ∼0.68 instead predict an exponent of ∼0.75 in agreement with “Kleiber’s law”, and this value was subsequently recapitulated in a separate analysis [28]. This discussion also complements the concepts that *1*) intra-species REE and inter-species REE scale to 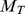 with different allometric scaling exponents [26] and *2) “that no single relationship is appropriate for describing the relationship between MR [metabolic rate] and M for all mammals, and that relationships for more narrow taxonomic groups or body mass ranges should be used when predicting MR from M”* [38].

### Future directions

Progress toward a richer mechanistic understanding of the biobehavioral regulation of whole-body EE in health and disease will benefit from developments in the realms of mathematical modeling and basic biology. Because EE scales in accordance with power equations, both at the whole-body and at the individual organ-tissue level, EE modelers should give greater consideration to the development of multi-tissue compartment allometric models (for examples involving lean and fat mass [46], [47]). Allometric EE model development will, of course, entail study designs that involve broad ranges of body mass and composition, as well as relatively large sample sizes. In return, multi-organ allometric models may confer novel mechanistic and practical insights, and will possess the aesthetic and possibly practical advantages of having true zero y-intercept values, a goal that will remain elusive with multi-organ linear models if the analysis presented herein is correct.

Advances in EE analysis will require a better understanding of the within species scaling of individual organ mass-specific metabolic rate (both at the levels of REE and average daily EE) in relation to body mass and composition. Although a few published studies on this topic suggest that adult within-human mass-specific organ-tissue REE is relatively constant across body size and adiposity [58]–[61], a great deal remains to be learned about organ mass-specific metabolic rate in diverse states of disease and neuroendocrine status. It seems particularly important to stress that almost nothing is known about the scaling or regulation of organ mass-specific metabolic rate in mice, which for better or worse hold immense sway over the conduct and direction of human biomedical research.

## Supporting Information

Appendix S1 **Proof of concept and R code used for Monte Carlo simulations.**
(DOC)
