# Supplementary material for: Mathematical Model for the Contribution of Individual Organs to Non-Zero Y-Intercepts in Single and Multi-Compartment Linear Models of Whole-Body Energy Expenditure
Source: PLoS One. 2014 Jul 28;9(7):e103301. doi: 10.1371/journal.pone.0103301 (PMC4113365; doi:10.1371/journal.pone.0103301)
Supplement: Appendix S1 — Proof of concept and R code used for Monte Carlo simulations. (DOC) [file pone.0103301.s001.doc]

**Online Supplement S1 for *Mathematical Model for the Contribution of Individual Organs to Non-Zero Y-Intercepts in Single and Multi-Compartment Linear Models of Whole-Body Energy Expenditure* by Karl J. Kaiyala, PhD**

Contents:

Proof of concept

R Code for Monte Carlo simulations

**Proof of concept** The purpose of this supplement is to *1)* demonstrate that the regression model$REE=a+bM_{T}$ is a ‘reasonable’ estimate of the first-order Taylor series $REE\approx cM_{0}^{k}(1-k)+kcM_{0}^{(k-1)}M_{T}$ for the true population model$REE=cM_{T}^{k}$ and *2)* that Eqs. 6 and 7 in the manuscript do provide ‘reasonable’ estimates of $k\text{ and }c$. In the process it was natural to generate estimates of $k\text{ and }c$ from the standard method for comparison with the new method (the standard method regresses $\log(EE)$ on $\log(M_{T})$. The slope estimate for $\log(M_{T})$ is the estimated $k$ value and the antilog of the intercept term is the estimated $c$ value ).

I wrote a Monte Carlo simulation program in R (version 3.0.0; R Core Team (2013). R: A language and environment for statistical computing. R Foundation for Statistical Computing, Vienna, Austria. URL <http://www.R-project.org/>). The R code is given below. Simulations assumed a population allometric model as follows: $REE=0.5M_{T}^{\text{ }0.67}e^{\varepsilon}$where the parameter $\varepsilon$ denotes normally distributed random error with mean zero and standard deviation $\sigma_{\log}$ (i.e., ) on the log scale (termed log-normal error). Accordingly, the factor $e^{\varepsilon}$is a multiplicative error term that scales error in proportion to the expected value of the dependent variable. This represents the dominant error model in allometric analysis . However, predicting the parameters generated by the multiplicative error model from an analysis involving the additive error model (where $\sigma_{Linear}$ is the standard deviation of the random error for the linear model) raises questions about bias in those parameter estimates. A first approach to examining bias in the parameter estimates was to generate estimates that represent the parameter values achieved in a restricted range analysis as the sample size grows enormous. To do so, the sample size was specified as n=100,000 and $\sigma$was set at 0.05.

Figure S1 depicts the 100,000 simulated REE values randomly drawn from the population allometric equation for a restricted range of $M_{T}=30\pm2\text{ g}$(all data are reported as mean $\pm$ SD). The parameter estimates of the linear regression in Figure S1 equal (to two significant decimal places) those predicted by the first-order Taylor linearization of the population allometric equation in depicted in Figure 1 of the manuscript. The estimated $k\text{ and }c$ values from the new and standard methods were both 0.67 and 0.50.

To examine whether estimates were sensitive to the value of $M_{T}$ on which the restricted range linear analysis was centered, I repeated this simulation for values of $M_{T}=10,\text{ }20\text{ and }40\pm2\text{ }g$ with all other parameters as indicated above. In each case, the y-intercept and slope parameters that were estimated from the linear regression agreed well with those predicted by the first-order Taylor linearization (difference <3.2% for the intercept and < 1.1% for the slope). In each case, the estimated $k\text{ and }c$ values, respectively, from the new and standard methods were both 0.67 and 0.50.

To examine the performance of the new vs. standard methods in settings involving a modest sample size (n=50), I ran 5000 simulations using the same population parameters as above and with $M_{T}=30\pm2\text{ g}$. The mean r-square of the linear fits was 0.45. The mean $a$ estimate of the fits was 1.61 $\pm$ 0.528 while the mean $b$ estimate was 0.11$\pm$0.018. Both the new and standard methods identified mean $k$ estimates of 0.67 $\pm$ 0.107. The mean $c$ estimates from the new and standard methods were 0.54 $\pm$ 0.205 vs. 0.54$\pm$0.204, respectively. Note that the $c$ estimates (but not the $k$ estimates) departed from the true population value of 0.50 with the reduction in sample size, but importantly both methods produced nearly identical estimates. Further analysis indicated that mean $c$ (but not $k$) estimates became larger as sample sizes decreased. Importantly, however, both the new and standard methods produced very similar $c$ estimates. The tendency for inflated $c$ values with lower sample sizes occurred because distributions for this parameter estimate were right skewed (medians were very close to the population value of 0.5), whereas $k$ estimates were always normally distributed with point estimates that were very close to the true population value of 0.67. The estimates for $a$ and $b$ were normally distributed.

These simulations demonstrate that despite the differences in the allometric and linear error models, the new method based on restricted range linear regression results in parameter estimates that are in good agreement with those of the true population allometric equation and its first order Taylor linearization. More sophisticated empirical and theoretical analyses involving parameter bias are certainly possible and may be of interest.

**Figure S1**

| 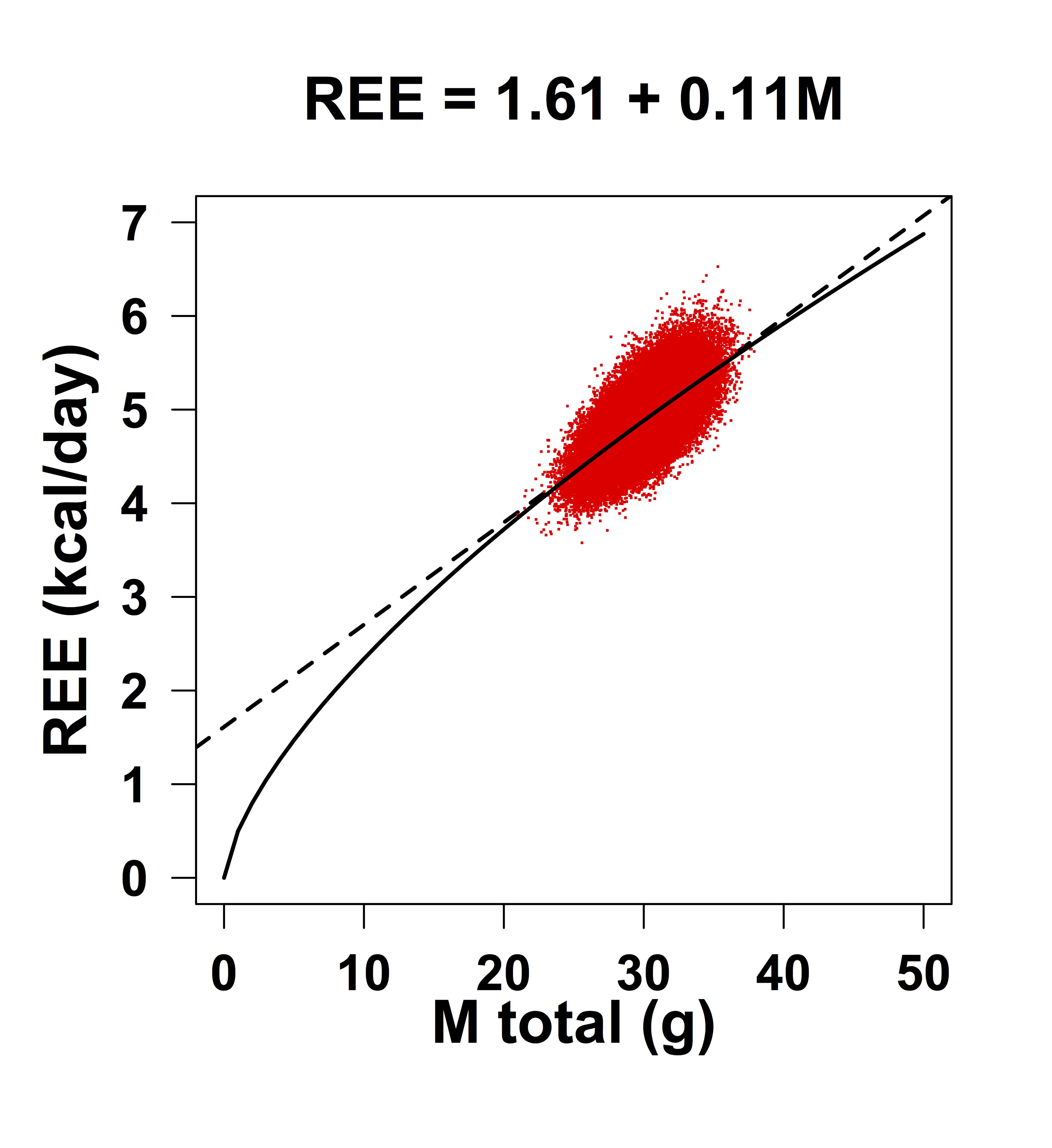 |
| --- |

**Figure S1 legend**. Example of a linear regression fit (r^2^ = 0.44) to restricted $M_{T}$-range data having a mean $\bar{M}_{T}$of $30\pm2\text{ (SD) }g$. The n=100,00 data points were randomly drawn using a Monte Carlo simulation from the population allometric EE function $EE=0.5M_{T}^{\text{ }0.67}e^{\varepsilon}$where . The parameter estimates of the linear fit are congruent with the y-intercept and slope of the allometric equation’s first-order Taylor series about $M_{0}=\bar{M}_{T}=30g$. To convert the allometric scaling coefficient to $\frac{kcal}{d\cdot(kg)^{k}}$, multiply by $(1000)^{k}$. To convert the slope of the Taylor series to units of $\frac{kcal}{d\cdot kg}$, multiply by 1000; the intercept remains unchanged.

**R Code for running simulations (annotated)**

nsims<-1000 #number of simulations

n0<-50 #sample size

m0<-30 # mean body mass of each sample

sd0<-2 #standard deviation of body mass

c<-.5 #allometric scaling coefficient

k<-.67 #allometric scaling exponent

z<-.05 #sigma for multiplicative error term for the "true" allometric population EE model; The true allometric EE equation is a power function according to which EE = powfun= (c*M^k)*exp(rnorm(n=n0,mean=0,sd=z)) as specified below.

k.log<-rep(NA,nsims) #k estimated from sampling k estimates from log-log fits to sampled data

k.lin<-rep(NA,nsims) #k estimated from linear regression fits to sampled data

beta<-rep(NA,nsims)# slope estimate for linear regression of EE on M

yint<-rep(NA,nsims)#y-intercept for linear regression of EE on M

c.lin<-rep(NA,nsims) #c estimated from linear regression fits to sample data

c.log<-rep(NA,nsims) #c estimated from log-log fits to sampled data

r.sqEE<-rep(NA,nsims) # r-square of the linear fits to sampled data

dk.lin_k.log<-rep(NA,nsims)# difference between k estimates between k estimates from linear fits and log-log fits computed as k.lin minus k.log

EE.atmeanM<-rep(NA,nsims)#EE computed at mean M using paramters for population EE model but excluding the multiplicative error term

meanEE<-rep(NA,nsims)

meanM<-rep(NA,nsims)

deltaEE<-rep(NA,nsims)

for(i in 1:nsims) {

M<-rep(rnorm(n=n0,mean=m0,sd=sd0))

powfun<-(c*M^k)*exp(rnorm(n=n0,mean=0,sd=z)) #population power function from which EE data are sampled

#powfun<-(c*M^k)+rnorm(n=n0,mean=0,sd=z)*5

EE<-powfun # rename output from power function 'EE'

linfit<-lm(EE~M) #linear regression fit to EE data

loglogfit<-lm(log(EE)~log(M)) #log-log fit to data. log = natural log in r

k.lin[i]<-summary(linfit)$coefficients["M","Estimate"]*mean(M)/(summary(linfit)$coefficients["M","Estimate"]*mean(M)+summary(linfit)$coefficients["(Intercept)","Estimate"]) # calculates scaling exponent k from linear regression fit

beta[i]<-summary(linfit)$coefficients["M","Estimate"]# slope parameter for linear fit

yint[i]<-summary(linfit)$coefficients["(Intercept)","Estimate"]# y-intercept parameter for linear fit

k.log[i]<-summary(loglogfit)$coefficients["log(M)","Estimate"] # calculates scaling coefficient from log-log fit

c.log[i]<-exp(summary(loglogfit)$coefficients["(Intercept)","Estimate"])

meanEE[i]<-mean(EE)

meanM[i]<-mean(M)

c.lin<-meanEE/meanM^k.lin # allometric scaling coefficient est. from linear fit

r.sqEE[i]<-summary(linfit)$r.squared # gets r-squared value from linear regression fit

}

mean(k.lin)

var(k.lin)

sd(k.lin)

mean(c.lin)

var(c.lin)

sd(c.lin)

mean(k.log)

var(k.log)

sd(k.log)

mean(c.log)

var(c.log)

sd(c.log)

mean(r.sqEE)

mean(deltaEE)

hist(k.lin)

hist(k.log)

hist(c.lin)

hist(c.log)

x<-c(0:50)

poppowfun<-c*x^k # produces error-free EE for "latent" population allometric EE curve

y<-poppowfun # assigns 'y' to population power function

#plot(M,EE,xlab="M (g)",ylab="REE (kcal/day)",font.lab=2,font.axis=2,pch=20,las=1,mgp = c(2.1,1,0),xlim=c(0,50),ylim=c(0,7),abline(lm(EE~M)))

#lines(x,y)

#title(main="B",adj=0)#adj=0 left justifies

plot(M,EE,xlab="M total (g)",ylab="REE (kcal/day)",font.lab=2,font.axis=2,pch=".",col="red",las=1,mgp = c(2.1,1,0),cex.lab=1.5,cex.axis=1.25,xlim=c(0,50),ylim=c(0,7),abline(lm(EE~M),lwd=2,lty=2))

lines(x,y,lwd=2)

title(main="REE = 1.61 + 0.11M",cex.main=1.5,adj=.5)

#axis(side=1,lwd=2)

#axis(side=2,lwd=2)

#plot(M,EE,xlab=expression(M (g)),ylab="REE (kcal/day)",font.lab=2,font.axis=2,pch=20,las=1,mgp =c(2.5,1,0),cex.lab=1.5,cex.axis=1.25,xlim=c(0,50),ylim=c(0,7),abline(lm(EE~M),lwd=3,lty=2))

#lines(x,y,lwd=3,col="black")

#title(main=expression(0.5*M[T]^0.67*e^epsilon) ,cex.main=1.5,adj=.5)

#title(main="B",cex.main=1.5,adj=0)

#mean(mean(EE)/mean(M)^k.lin)#for unkown reason works to give c.lin if paste post hoc but not apriori

#c.lin<-mean(EE)/mean(M)^k.lin

#mean(c.lin)

#var(c.lin)

#sd(c.lin)

#hist(c.lin)

mean(beta)

sd(beta)

mean(yint)

sd(yint)

summary(linfit)

**Supplemental References**

1. Xiao X, White EP, Hooten MB, Durham SL (2011) On the use of log-transformation vs. nonlinear regression for analyzing biological power laws. Ecology 92: 1887-1894.

2. Lighton JRB (2008) *Measuring Metabolic Rates: A Manual for Scientists*. New York, NY, Oxford Univ. Press.

3. Lai J, Yang B, Lin D, Kerkhoff AJ, Ma K (2013) The allometry of coarse root biomass: log-transformed linear regression or nonlinear regression? PLoS One 8: e77007.
